# Biology of high single doses of IORT: RBE, 5 R’s, and other biological aspects

**DOI:** 10.1186/s13014-016-0750-3

**Published:** 2017-01-19

**Authors:** Carsten Herskind, Lin Ma, Qi Liu, Bo Zhang, Frank Schneider, Marlon R. Veldwijk, Frederik Wenz

**Affiliations:** 1 Department of Radiation Oncology, Universitätsmedizin Mannheim, Medical Faculty Mannheim, Heidelberg University, Theodor-Kutzer-Ufer 1-3, 68167 Mannheim, Germany; 2 Present Address: Department of Radiation Oncology, University of California, San Francisco, CA USA; 3 Present Address: Department of Oncology at No. 2 Hospital Anhui Medical University, and School of Life Sciences, Anhui Medical University, Hefei, Anhui China

## Abstract

Intraoperative radiotherapy differs from conventional, fractionated radiotherapy in several aspects that may influence its biological effect. The radiation quality influences the relative biologic effectiveness (RBE), and the role of the five R’s of radiotherapy (reassortment, repair, reoxygenation, repopulation, radiosensitivity) is different. Furthermore, putative special biological effects and the small volume receiving a high single dose may be important. The present review focuses on RBE, repair, and repopulation, and gives an overview of the other factors that potentially contribute to the efficacy. The increased RBE should be taken into account for low-energy X-rays while evidence of RBE < 1 for high-energy electrons at higher doses is presented. Various evidence supports a hypothesis that saturation of the primary DNA double-strand break (DSB) repair mechanisms leads to increasing use of an error-prone backup repair system leading to genomic instability that may contribute to inactivate tumour cells at high single doses. Furthermore, the elimination of repopulation of residual tumour cells in the tumour bed implies that some patients are likely to have very few residual tumour cells which may be cured even by low doses to the tumour bed. The highly localised dose distribution of IORT has the potential to inactivate tumour cells while sparing normal tissue by minimising the volume exposed to high doses. Whether special effects of high single doses also contribute to the efficacy will require further experimental and clinical studies.

**Electronic supplementary material:**

The online version of this article (doi:10.1186/s13014-016-0750-3) contains supplementary material, which is available to authorized users.

## Background

Technological advances in mobile radiotherapy (RT) units have greatly increased the clinical application of intraoperative radiotherapy (IORT) [1–4] by providing highly localized beams of low-energy X-rays (LEX) or high-energy (MeV) electrons. The physical radiation qualities of these sources may differ to some extent from that of high-energy photons (MeV X- or γ-rays) used in conventional external beam RT, and thus potentially influence the relative biologic effectiveness (RBE). Similarly, differences in dose distributions will influence the biological effects on residual tumour cells after excision of the tumour and on normal tissue cells in the tumor bed. Furthermore, delivering the total dose of adjuvant RT in a single large fraction is a major departure from conventional fractionated external beam RT with typical daily fraction sizes of 1.8–2.0 Gy. The rationale for fractionated RT is based on the four R’s of RT: reassortment, repair, reoxygenation, and repopulation [5], to which radiosensitivity was later added [6]. Reassortment between fractions redistributes surviving cells over cell-cycle phases thus avoiding repeated irradiation in resistant phases. Repair influences the biological effects of dose rate and time between fractions. Reoxygenation is important for tumours containing acutely hypoxic fractions of malignant cells. Repopulation decreases the biological effect of RT with long overall treatment time, and the radiosensitivity of tumour cells to different single doses (i.e., the dose response) determines the biological effect when the fraction size is increased. In addition, very large dose fractions may induce effects at cellular, tissue, and systemic, levels that are different from those seen after fractionated schemes with moderate fraction sizes (1–3 Gy). Finally, the dose distribution influences the biological effect on tumour cells and normal tissue. Here we discuss these factors with an emphasis on the biological effects of radiation quality, repair, and the role of repopulation.

### Radiation quality

Currently, IORT is applied using isotropic fields of 50 kV X-rays or dedicated linear accelerators with parallel electron beams of nominally 3–12 MeV [7]. The radiation quality is characterized by the ionisation density which is quantified by the linear energy transfer (LET) [8]. Electrons and X-rays are low-LET radiations as opposed to α-particles and heavy ions which are high-LET radiations. The LET increases with decreasing energy and thus the LET of LEX is higher than that of high-energy electrons although both are low-LET radiations with LET values 1–2 orders of magnitude lower than that of high-LET radiation. [8–10]. LEX deposit a higher proportion of their energy as electron track ends with low energies (<1 keV) compared with high-energy X-rays. Thus LEX will produce more lethal DNA lesions (double-strand breaks, DSBs, and complex lesions) per Gy resulting in an increased RBE [9–12].

The RBE of 50 kV X-rays from the Intrabeam® system (Carl Zeiss Meditec AG, Jena, Germany) for cell inactivation in vitro was determined for irradiation in a tumour-bed phantom. Irradiation at a distance of 8 mm from the surface of a 4 cm spherical tumour-bed applicator showed significantly increased RBE values relative to the reference radiation of 6MV X-rays [13]. The RBE values were comparable with that of a 50 kV surface X-ray unit and various published studies using experimental LEX sources [14–17] but was lower than the experimental values reported for the Intrabeam predecessor source operated at 40 kV X-ray without an applicator [18].

The RBE is defined as the dose ratio of the reference and test radiations producing the same biological effect: RBE = D_ref_/D_test_. In terms of the linear-quadratic (L-Q) formalism ln(SF) = −(α × D + β × D^2^) where SF is the surviving fraction of cells, D is the single-fraction dose, and α and β are the linear and quadratic coefficients, this implies that RBE → α_test_/α_ref_ in the low-dose limit (D → 0 Gy) while the high-dose asymptotic limit will be RBE → 1 if the value of β is the same for the two radiations. Thus, for high-LET radiation and LEX with RBE > 1, the L-Q formalism predicts a maximum RBE value at D = 0 Gy and a decrease in RBE with increasing dose. However, Liu et al. [13] found no significant dependence of RBE on dose indicating an effect of RBE on both the linear and quadratic components of the linear-quadratic model. This agrees with previous RBE studies on low-LET radiations [14–17] but contrasts with the assumptions of the L-Q model that the radiation quality affects mainly the linear term representing irreparable lesions [19, 20]. On the other hand, a study on monoenergetic 8 keV photons was consistent with the L-Q assumption of an effect on the linear component and showed further radiobiological effects reminiscent of high LET [21]. The latter is unexpected because photoelectrons released by interactions of photons with water and other molecules in the cells should not be different from track-ends of electrons with higher initial kinetic energy. These apparently conflicting result might be reconciled if a proportion of the absorption events of 8 keV photons result in emission of highly localized low-energy Auger electrons that produce more complex damage [22–24].

The unfiltered energy spectrum from Intrabeam includes a substantial contribution of low photon energies which are filtered over the first 1–2 cm of water-equivalent material resulting in hardening of the beam within the spherical applicators [9, 25]. Whether further beam hardening occurs in the tumour bed targeted by IORT is unclear but attenuation of the radial dose function for 50 kV X-rays is nearly constant at 2.0–3.5 cm radial distance from the source suggesting no gross change in radiation quality [9].

**Fig. 1:**
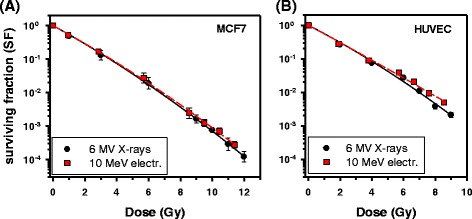
Survival *curves* for irradiation of cells in vitro with 10 MeV electrons (20 mm water-equivalent depth) or 6 MV X-rays. The RBE of electrons was not significantly different from 1 (RBE = 0.98 ± 0.01; *P* = 0.10, *n* = 3) for MCF7 cells (**a**) while RBE was significantly reduced (RBE = 0.91 ± 0.02, *P* = 0.015, *n* = 3) after irradiation of HUVEC with higher doses (**b**). These experiments corroborated trends observed in independent experiments at lower doses (Additional file 2: Figures S1B, C)

Because the energy of MeV electron beams is usually higher than the mean energy of secondary electrons produced when 6MV X-rays interact with water or tissue, the RBE of high-energy electrons may be slightly lower than that of X-rays. RBE values of 0.9 ± 0.1 for 11 MeV electrons relative to ^60^Co γ-rays (E = 1.25 MeV) have been published [17] but the RBE relative to 6MV X-rays used in modern external beam RT has not been determined so far. Therefore, we measured the RBE of 10 MeV electrons from a linear accelerator for cell survival in vitro. Survival of V79 cells showed no difference between 10 MeV electrons and 6MV X-rays at doses up to 6–8 Gy but an increase of surviving fractions (SF) was suggested in the dose range 10–12 Gy (Additional file 1: Supplementary Material and Additional file 2: Figure S1A). This trend was confirmed in independent experiments where the dose was extended to 14.3–17.1 Gy yielding an RBE value of 0.94 ± 0.02 (*P* = 0.04, *n*=3) at SF = 0.0003. For MCF7 breast cancer cells (Fig. 1a, Additional file 2: Figure S1B) no significant difference was observed up to 11.4 Gy (RBE = 0.98 ± 0.01, *P* = 0.10, *n*=3) at SF = 0.0003 but normal human umbilical vein endothelial cells (HUVECs; Fig. 1b and Additional file 2: Figure S1C) showed significantly decreased values of RBE = 0.93 ± 0.02 (*P* = 0.005, *n* = 6) at SF = 0.03 (mean electron dose 5.8 Gy) and RBE = 0.91 ± 0.02 (*P* = 0.015, *n* = 3) at SF = 0.005 (mean electron dose 8.7 Gy). However, the data seemed to indicate an effect on the quadratic term which is considered to represent potentially lethal but reparable lesions [26]. Thus, overall the RBE of 10 MeV electrons was only moderately or not significantly reduced relative to 6MV X-rays in the three cell lines tested. This supports clinical practice from fractionated RT assuming RBE = 1 for electrons given in standard fraction sizes. The fact that a reduced RBE was only detected at higher doses may either simply reflect the different slopes of the survival curves or possibly indicate a role of reparable damage in the RBE of low-LET radiations. Whether the apparent difference between MCF7 and HUVEC is characteristic for tumour and normal cells will require further studies.

It is important to note that differences in the RBE of low-LET radiations with different beam energies do not influence the *quality* of different IORT modalities *per se*. Although the yield of lethal lesions per Gy will differ, doses from different radiation qualities may be compared by multiplying the physical dose with the RBE to give the isoeffective dose of the reference radiation. However, the types of lesions are the same since the ionisation tracks are produced by the same kind of particles, namely primary or secondary electrons. This contrasts with high-LET radiations such as C-ions, which produce a dense track of ionisations resulting in mainly complex, irreparable lesions.

### Reassortment

Cellular radiosensitivity varies through the cell cycle, with mitosis and late G1 phase being sensitive while the late synthetic (S)-phase is relatively resistant [8]. Thus surviving cells after irradiation of asynchronous cell populations will be enriched in the more resistant phases. During fractionated radiotherapy of tumours with rapid cell proliferation, heterogeneity in cell cycle kinetics will redistribute (reassort) cells over the cell cycle between daily fractions [27]. Obviously, reassortment does not play a role in IORT with a single dose. However, single-dose cell survival curves show no evidence of a resistant subpopulation which should manifest itself by a decreased slope at higher doses similar to that observed for hypoxic subpopulations [8]. Obviously the increased efficacy of incremental doses suffices to compensate the increase in radioresistance caused by the stronger inactivation of radiosensitive cell-cycle phases. Some potential mechanisms contributing to high-dose radiosensitivity will be discussed below.

### Repair – dose dependence

While the induction of DSBs is proportional to dose, the repair system may conceivably become saturated at higher doses. Saturated repair has been suggested to explain the downward curvature of low-LET cell survival curves [28, 29]. Mammalian cells repair DSBs mainly by non-homologous end-joining (NHEJ) which is the primary DSB repair mechanism in all cell-cycle phases and rejoins double-stranded DNA ends without requirement for homology [30, 31]. A smaller fraction of DSBs is repaired by homologous recombination (HR) which is error free but requires a sister chromatid strand as template and thus is active only in late S and G2 [30, 32, 33]. Rejoining of ‘simple’ DSBs in euchromatin is performed by NHEJ with fast kinetics while DSBs in heterochromatin, and complex DSBs which failed to be repaired by NHEJ, are repaired by HR with slow kinetics [30, 34, 35].

**Fig. 2:**
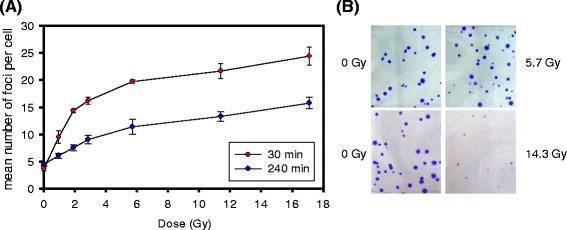
Sublinear dose response for the mean number of γH2AX foci per cell (V79) at maximum induction and after 4 h repair (30 min and 240 min post-irradiation, respectively) (**a**). Reduced colony size of V79 cells irradiated 14.3 Gy but not 5.7 Gy (10 MeV electrons, 20 mm water-equivalent depth) (**b**)

Induction and repair of DSB can be monitored by antibodies against phosphorylated histone γH2AX which marks DSB sites and acts as scaffold for the DSB repair machinery. Induction of γH2AX foci occurs within minutes after irradiation and reaches its maximum at approximately 30 min (Additional file 1: Supplementary Material and Additional file 2: Figure S2A). This method can detect DSBs after doses in the range 0.001–2 Gy and showed similar yields of foci per Gy as for physical DSBs measured in the range 10–100 Gy in human fibroblasts [36]. However, a sub-linear increase in the number of γH2AX foci at doses higher than 2–3 Gy has been described for different cell lines [37, 38]. This did not appear to be caused by overlapping foci imposing an upper limit for detection of individual foci. First, cell types with different yields of foci per Gy showed similar sub-linearity even at dose levels where foci were not overlapping. [38, 39]. Second, the distribution of foci in individual cells was not skewed towards high numbers as expected if an upper limit is reached (Additional file 2: Figure S2B-E). Third, the deviation from linearity 240 min after irradiation was observed at approximately the same dose as at 30 min, in spite of a much lower mean number of foci after repair (Fig. 2a). Similar observations were made with MCF7 and HUVECs (Additional file 2: Figure S3A, B), and with human skin fibroblasts (Herskind et al., manuscript in preparation). Further evidence indicated that the fraction of remaining foci was lower at low dose and increased with dose, suggesting that the rate of foci decay is reduced at higher doses (Additional file 2: Figure S3C). This supports the hypothesis that a saturated repair process, rather than optical overlap of foci, is involved in the non-linear dose response.

Most studies have found DSBs to be repaired with bi-exponential kinetics representing the fast and slow components, although a third, even faster component with halftime <5 min has been described [40, 41]. Physical methods for measuring DSBs have shown a high capacity for fast rejoining which starts immediately after irradiation and repairs more than 50% of the DSBs before induction of γH2AX foci reaches its maximum [42–45]. This might be explained if the foci are too small to be detected by immunofluorescence microscopy or are quickly resolved [40, 42, 46]. However, high-resolution studies using transmission electron microscopy (TEM) showed that NHEJ-mediated repair of DSBs (marked by pairs of Ku70 protein binding to the DNA double-strand ends) occurred with fast kinetics in euchromatin and was not associated with DNA repair foci in contrast with DSBs in heterochromatin which were associated with DNA repair foci and were repaired with slow kinetics [47, 48].

Further evidence showed that foci may combine with neighbouring foci at distances up to 1–2 μm indicating the formation of repair centres [49]. This may explain a previous observation that the linear range of the γH2AX dose response at low doses was extended to higher doses before the transition to sublinearity when the integrated fluorescence intensity was detected by flow cytometry compared with microscopic counting of foci numbers [38]. The decay of γH2AX foci after 30 min could be fitted by bi-exponential kinetics although the data were also compatible with a hyperbolic fit (Additional file 2: Figure S4). The decay of γH2AX foci usually occurs more slowly than physical DSB repair, which has been suggested to be related to limited phosphatase activity required for their resolution, and to foci in heterochromatin which are formed and resolved more slowly than in euchromatin [40, 42]. The observation from TEM studies that some foci remained at late times without evidence of DSBs suggested that they may mark epigenetic changes in chromatin structure [47]. Nevertheless, it also seems possible that repair centres processing several DSBs will persist until the last local DSB is repaired, and thus decay more slowly than expected from the repair of individual DSBs. The notion of repair centres would seem consistent with the observation of foci containing more than a single DSB in TEM studies [47, 48].

**Fig. 3:**
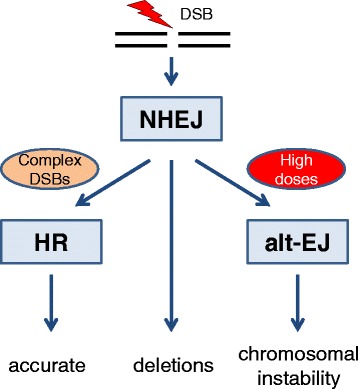
Proposed schematic model of increasing use of alternative end joining (alt-EJ) leading to increased chromosomal instability at higher doses. HR: homologous recombination. NHEJ: non-homologous end joining. Modified after Shibata and Jeggo [30]

Formation of repair centres would contribute to reduce the number of foci (though not their integrated intensity) at 30 min. If availability of the DSB repair machinery is limited, this might conceivably impose a limit on the rate at which DSBs can be processed. Indeed, previous evidence suggested that HR is saturated at high doses with an increasing majority of DSBs being repaired by NHEJ [33]. An error-prone alternative end joining (alt-EJ) pathway has been proposed to act as a backup repair mechanism for NHEJ [50, 51]. In contrast with classical NHEJ, in which the Ku70/Ku80 heterodimer and DNA-PKcs stabilise the DSB ends which are then processed and finally ligated by LIG4/XRCC4, alt-EJ uses proteins otherwise involved in DNA metabolism. Thus PARP1 and WRN stabilise the free ends which are ligated by LIG3/XRCC1 or LIG1 after processing of the ends [50]. Cumulative evidence supports the view that this does not represent a distinct DSB repair pathway but rather a means of removing free DNA ends left unrepaired by NHEJ and HR [50]. Furthermore, alt-EJ is associated with increased chromosome translocations which are normally suppressed by NHEJ [51–53]. Thus, in the present context, we propose that saturation of HR and overloading of the NHEJ pathway result in increased use of the alt-EJ pathway and increased chromosomal instability at higher doses. The small size of colonies formed after 14.3 Gy but not 5.7 Gy may indicate genetic instability of surviving cells after high doses (Fig. 2b). A model of changing DSB repair pathway usage at high single doses is summarised in Fig. 3.

### Repair – protracted irradiation and fractionation

Recovery of cells between fractions is an important factor in fractionated RT. When irradiation of cells is split into two doses, e.g., a fixed dose D1 and a variable dose D2 separated by a time interval, T, the surviving fraction (SF) will be higher than when given as a single dose, D1 + D2. If the time interval is increased to allow complete repair, the shape of the survival curve for the second irradiation will approach that of the single-dose survival curve starting at a lower survival level, SF(D1). Frequently, such split-dose recovery is ascribed to repair of socalled ‘sublethal damage’ (SLD) [8]. Continuous irradiation with constant dose rate may be viewed as multiple small fractions given at short intervals thus providing the basis for the reduced biological effect of protracted irradiation. The biological effect of incomplete recovery between fractions or continuous low dose-rate irradiation can be calculated using mathematical models assuming monoexponential SLD repair [26, 54, 55]. In order to account for a decreasing rate of SLD repair, reciprocal recovery kinetics has been proposed, which has the advantage that just two free parameters need to be fitted compared with four parameters of a bi-exponential model [56, 57], thus providing a more robust fit requiring fewer data points.

Although DSB repair by HR or NHEJ has been implicated in SLD repair [58–61], the relative importance of the two mechanisms, and their relation to the biophysical models, is not clear. For normal tissue, recovery kinetics derived from fractionation studies show halftimes of approximately one hour or longer [62]. However, early normal-tissue reaction in experimental systems yielded shorter halftimes of 0.3–0.8 h [63], and some clinical and experimental studies on early and late reaction showed biphasic recovery with halftimes of 7–20 min and 1.2–6.6 h for the fast and slow components, respectively [64–67]. For tumour cells, most evidence suggests a single-component halftime of the order of one hour [62]. However, other studies have found shorter recovery halftimes in the range 5–50 min [68] or 23 min with some evidence of bi-exponential recovery with halftimes of 18 and 96 min [69].

For IORT given as protracted irradiation with Intrabeam, the L-Q model predicts that SLD repair should reduce the biological effect of higher doses [9, 70]. Since protracted IORT with Intrabeam typically requires 20–50 min, only recovery within this post-irradiation time frame is relevant. Split-dose experiment with V79 hamster and MCF7 human breast cancer cells yielded halftimes of 15 min and 39 min, respectively (Additional file 1: Supplementary Material and Additional file 2: Figure S5). For V79 cells, a slower rate of repair was indicated after 1–2 halftimes. Previous modelling calculations of the biological effect of SLD repair for protracted irradiation with Intrabeam used the L-Q formalism and assumed halftimes of 15 min as a conservative limit for fast repair [25, 69–71]. The present values together with the evidence from literature supports the use of this conservative value and suggests that the effect of recovery may be smaller if halftimes are longer, although recovery halftimes are short enough relative to the irradiation times for IORT that they should be taken into account.

The L-Q formalism is used to model effects of changes in fractionation and dose rate. The underlying α/β parameter describes the downward curvature of single-cell survival curve and the sensitivity to changes in fraction size [63]. However, it should be noted that α/β for fractionation is determined from fractionation studies with full recovery between daily fractions, and not from cell survival curves. For local control after breast RT, the most recent estimate is α/β = 4 Gy (c.i. 0.0–8.9 Gy) at median 9.3 years follow-up with some evidence that it may even be slightly lower [72]. This is comparable with α/β for late normal-tissue (NT) reaction (shown in Table 1) leading to the conclusion that the therapeutic window between tumour control and adverse effects depends less on fraction size than previously assumed [73, 74].

**Table 1:** α/β ratios for different normal-tissue end points

End point	α/β	95% c.i.	Ref.
Breast shrinkage	3.5 Gy	0.7; 6.7 Gy	[72]
Breast induration	4 Gy	2.3; 5.6 Gy	[72]
Telangiectasia	3.8 Gy	1.8; 5.7 Gy	[72]
Breast oedema	4.7 Gy	2.4; 7.0 Gy	[72]
Skin fibrosis	1.7 Gy	0.6; 2.6 Gy	[146]
Telangiectasia	2.6–2.8 Gy	see references in	[147]
Pneumonitis	4.0 Gy	2.2; 5.8 Gy	[148]
Lung fibrosis (radiol.)	3.1 Gy	−0.2; 8,5 Gy	[149]

### Reoxygenation

Oxygen is a radiosensitizer of cells owing to its ability to fix DNA damage which may otherwise be chemically repaired by intracellular antioxidants under hypoxia [8]. Thus the cellular radiosensitivity is reduced under hypoxia. Some tumors contain chronically or acutely hypoxic subpopulations requiring 2- to 3-fold higher doses for inactivation [8]. Reoxygenation of acutely hypoxic cells between daily fractions is an important aspect of fractionated radiotherapy. In tumour-bed IORT, the bulk tumour has been removed by surgery and thus only microscopic clusters of tumour cells should be present in the healthy tumour bed. Under most conditions, it seems reasonable to assume that these cells will have an adequate supply of oxygen. However, care should be taken not to compress the tumour bed too strongly during IORT with LEX or otherwise compromise the blood supply to an extent that might induce hypoxia in the tumour-bed tissue.

### Repopulation

An important aspect of IORT is that irradiation takes place immediately after tumour excision thus avoiding repopulation of remaining tumour cells during the time between surgery and conventional external beam radiotherapy. Although EBRT should begin as soon as possible, a five-week period for wound healing is required before starting EBRT in order to avoid excessive normal-tissue reaction [75]. A systematic review showed that delaying EBRT for more than 8 weeks in breast cancer, and more than 6 weeks in head and neck cancer, resulted in increased recurrence rates, emphasizing the importance of repopulation after surgery in these tumours [76, 77]. Furthermore, in the START B fractionation trial, recurrence rates were reduced (though not significantly) when hypofractionated EBRT (15 × 2.67 Gy) was given over three weeks compared with five weeks in in the conventional arm (25 × 2.0 Gy, EQD2 = 50 Gy where EQD2 is the equivalent dose given in 2 Gy fractions) [72]. This observation was in spite of the equivalent total dose, EQD2, being lower in the accelerated, hypofractionated arm: EQD2 = 44.5 Gy if given in 2 Gy fractions (assuming α/β = 4 Gy). The efficacy of the shorter overall treatment time strongly suggests that repopulation between fractions plays an important role and could be equivalent to a daily dose of 5.5 Gy/14d = 0.4 Gy per day^1^. The increased recurrence rate observed for the post-pathology stratum in the TARGIT trials for patients treated some weeks after surgery compared with patients irradiated during the surgical session [78] may be explained by repopulation. Thus a major advantage of IORT for fast proliferating tumours seems to be the elimination of repopulation by the extreme shortening of the overall treatment time. Since conventionally fractionated EBRT cannot be completed earlier than ten weeks after surgery (five weeks delay after surgery plus five weeks of treatment), the associated benefit might potentially be equivalent to a dose of the order of 28 Gy (70d × 0.4 Gy/d). This might even be a conservative estimate as it assumes the same rate of repopulation during the delay as during RT, and does not include additional time for a tumour boost given after whole-breast RT. Further studies of the influence of overall treatment time in fractionated radiotherapy should be performed to validate this hypothesis and provide more accurate estimates of the dose equivalent of repopulation in different tumours.

### Radiosensitivity

The risk of recurrence after RT depends primarily on the number of surviving tumour cells capable of regrowing the tumour. According to the cancer stem cell hypothesis, only a subpopulation of tumour cells have unlimited capacity for proliferation and it is generally considered that these cells are more resistant to radiation [79]. Cellular radiosensitivity is determined by the colony formation assay (CFA) which scores the ability of cells to produce clones with at least 50 cells corresponding to > 5–6 cell doublings. Although the CFA is the gold standard for determining radiosensitivity in vitro, detection of SF values <10^−3^ is notoriously difficult and ultimately is limited by the number of cells which can reasonably be seeded in the assay. In most cases the conditions of the assay will not be constant, either because increasing numbers of cells per flask or dish are seeded to keep the number of colonies constant with increasing dose, or because more cells are produced during incubation of unirradiated compared with irradiated cultures, in the case where constant numbers are seeded and the number of colonies decreases with increasing dose.

Although it is usually assumed that surviving colony-forming cells are identical to unirradiated cells, this may not be true in genetically instable tumour cells irradiated with high doses. As argued above, high doses of radiation may induce increased genetic instability, and certainly the colonies scored at high doses are morphologically different from those formed in unirradiated cultures. Thus genetic instability may influence not only the number but also the properties of surviving cells. Furthermore, the shape of the survival curve at high doses may be influenced by the number of cells seeded in the CFA, suggesting that non-targeted cohort effects play a role in cell inactivation by high doses [80].

Extrapolation of SF data to high doses used in IORT or stereotactic radiosurgery has been a matter of debate. It has been argued that cell survival curves have a linear slope at high doses and various modifications of the L-Q model, or alternative models, have been proposed to account for this [81–85]. On the other hand, there is evidence that the L-Q model fits quite well up to doses of approximately 15 Gy [86] and, despite trends in goodness of fit to the experimental data, a significant difference between fitting the L-Q model and alternative models has not been demonstrated [87–90]. In fact the values of fit parameters are at least as important as the choice of model for extrapolation [87, 91]. As argued above, various experimental and environmental factors may influence survival after high doses. Thus while tumour cell transplantation and in-vivo tumour-cell survival experiments reportedly produce linear survival slopes at high doses [92], actual survival levels in an IORT setting can only be estimated. A pragmatic approach, therefore, is to use the least complicated model with the lowest number of free parameters (e.g., the L-Q model) as a first approximation and closely monitor patients with an aim of establishing dose-response relationships from clinical data. It should also be noted that for non-uniform dose distributions such as the isotropic X-ray field from Intrabeam with its steep gradient, a 10% variation in dose only displaces the isodose curves by approximately 1 mm [13, 70].

### Other biological effects

In addition to clonogenic inactivation of tumour cells, RT excerts effects on the stroma, vasculature, and the immune system, that might influence the response of residual tumour cells. Radiation induces expression of inflammatory cytokines via NF-κB [93, 94], and cytokines in wound fluid collected from breast cancer patients treated with IORT were implicated in biological effects on migration and invasion of cancer cells [95] though it is unclear if proliferation was inhibited [96]. Furthermore, irradiation of endothelial cells can induce platelet adhesion and thrombus formation in the microvasculature [97, 98]. An overview of experimental studies on radiation-induced vascular damage was published recently [99].

A series of papers have implicated the second messenger ceramide in radiation-induced apoptosis of microvascular endothelial cells. Ceramide may be released from the membrane lipid sphingomyelin by the enzyme acid sphingo-myelinase (ASMase), and activation of ceramide synthase (CS) can lead to *de novo* synthesis of ceramide [100, 101]. ASMase-dependent apoptosis of ASMase - rich microvascular endothelial cells was proposed to constitute the primary target for radiation-induced intestinal damage after doses larger than 13–15 Gy while activated CS contributed to apoptosis at doses above 18–20 Gy [102, 103]. The protective effect of an anti-ceramide antibody strongly supported the proposed role of ceramide-induced endothelial damage in the radiation gastrointestinal syndrome [104]. Furthermore, endothelial apoptosis of the tumour vasculature was suggested to play an important role in radiation-induced tumour control after single doses of 15 Gy and more [105, 106]. Thus DNA damage-mediated inactivation of clonogenic normal or tumour stem cells (dominant at lower doses) and endothelial apoptosis (at higher doses) was proposed to constitute a two-target model for normal tissue damage and tumour inactivation after high doses per fraction [102, 103, 106, 107]. However, these findings have been disputed and remain controversial as they have not been reproduced in other laboratories [87, 108–110].

Radiation is an efficient modulator of the immune response and thus may have systemic effects that eventually help eliminate residual tumour cells. Higher radiation doses (>5 Gy) result in increased tumour cell necrosis and antigen presentation, and recruitment of T-cells to irradiated and possibly distant unirradiated tumours (for reviews see [9, 87]). In the setting of tumour-bed IORT, most patients will have no residual tumour cells while in a minority of patients the number of residual tumour cells will vary between very few and perhaps up to some 10^5^ cells in microscopic foci. Whether low numbers of tumour cells suffice to elicit an antitumour response, and whether a single high dose is more efficient than fractionated RT, is not known and will require more studies. Thus, in spite of a growing awareness of the importance of radiation in stimulating the immune system, it is not clear if it contributes to the efficacy of high single doses.

### Irradiated volume, local control and normal-tissue reaction

An important aspect of IORT, and more generally of accelerated partial breast irradiation (APBI), is the smaller volume exposed to high doses compared with whole-breast EBRT. The dose distribution varies between the different modalities for delivering IORT. Thus tumour-bed irradiation with LEX using spherical applicators to fit the cavity left by the excised tumour yields a non-uniform, isotropic dose distribution determined by a combination of the distance-squared relationship and beam attenuation. IORT with electrons (IOERT) delivers high-energy electrons in a parallel beam with a characteristic depth dose profile and range that depends on the chosen beam energy. A comparison of dose distributions from LEX, MeV electrons, and other IORT/APBI techniques, was published by Nairz et al. [111]. Conformality to the tumour bed may be an issue with both IORT techniques. For LEX, conformality depends on the fit of the tumour cavity to the spherical applicator, while the choice of applicator size and the incident angle is critical for covering the target volume treated by IOERT [111]. To treat other targets than the tumour bed around a spherical cavity with LEX, new Intrabeam applicators have been developed to irradiate intracavitary cylindrical targets and targets with flat geometries [112–115].

For the non-uniform dose distribution of isotropic LEX, radiobiological modelling of local control for tumour-bed IORT of the breast suggested that inactivation of recurrence-forming foci close to the applicator surface was more efficient than for external-beam radiotherapy (EBRT) with a uniform dose to the whole breast. The increased inactivation partly compensated the reduced inactivation at larger depth in the tumour bed thus defining a ‘Sphere of Equivalence’ within which the recurrence rate would be the same as for EBRT [71]. Nevertheless, the legitimate question arises whether doses applied by LEX are sufficient to control residual tumour cells at larger depth in the tumour bed where the physical dose is reduced to approximately 6–7 Gy in 1 cm depth and 2–3 Gy in 2 cm depth depending on the applicator size. By comparison, the dose distribution for IOERT is rather uniform up to the penetration depth determined by the beam energy and within the area covered by the applicator size outside which the dose decreases to virtually zero. However, after excision of the primary tumour the majority of patients develop no recurrences and the number of residual recurrence-forming tumour cells in patients who go on to develop local recurrence is likely to show wide variation ranging down to just a few cells. This implies that even low to moderate doses of radiation may contribute to local control as long as the tumour is excised with sufficient free margin leaving no solid tumour mass [9, 116].

The rationale of IORT for early breast cancer is based on studies showing that most ipsilateral recurrences occur close to the site of the primary tumour and that more distant recurrences may be considered new primary tumours with a more favourable prognosis [117–119]. Thus clinical studies testing the equivalence of IORT as sole treatment with conventional EBRT should be judged not only based on their dose distributions but should be considered a test of a hypothesis involving several elements: 1) local distribution of recurrence-forming residual tumour cells; 2) dose distribution; 3) single-dose versus fractionated irradiation; 4) total time between surgery and completion of RT; 5) patient selection. Comparing the TARGIT trial using non-uniform LEX and the ELIOT trial using IOERT, the former was a risk-adapted approach where low-risk patients were selected based on established risk factors and postoperative EBRT was added to the treatment based on the pathological findings. This constrasts with ELIOT in which unselected patients received IOERT with a nearly uniform dose distribution. Conformality of the cavity to the spherical applicator is important for dose coverage of the tumour bed in the TARGIT trial. In ELIOT, the choice of applicator size is critical for covering the target volume [120–123].

Both randomized trials were within the pre-defined non-inferiority margins for ipsilateral recurrence [78, 123]. However, increased recurrences were observed in the post-pathology IORT stratum of the TARGIT trial [78] suggesting that it is essential to perform IORT immediately after surgery and not delayed in a second surgical session. In ELIOT, recurrences in the IOERT arm were associated with unfavourable characteristics (tumour size ≥2 cm; ≥ 4 positive lymph nodes, grade 3 differentiation, estrogen receptor-negative and triple-negative tumours), suggesting that improved patient selection might reduce the recurrence rate after IOERT [123]. While differences in selection criteria, treatment strategies, and median follow up, hamper a direct comparison between the two IORT modalities, a critical analysis of the outcomes has been published [121, 122]. Excluding the unfavourable subgroups (pre-pathology treated patients in TARGIT, patients with unfavourable characteristics in ELIOT) yielded 2.1% local recurrence in TARGIT and 1.5% in ELIOT [78, 123]. Thus at present, there is no evidence to suggest that differences in the dose distributions affect the outcome.

IORT is frequently used as an intraoperative boost combined with conventional EBRT for breast conserving therapy. For IORT with non-uniform LEX, the full dose of IORT is applied while the total dose of EBRT is usually reduced slightly from 50 Gy to 46 Gy. For IOERT with uniform dose distribution, the IOERT dose is reduced to 8–10 Gy followed by standard EBRT (although in the ELIOT trial EBRT was given after full-dose IOERT in patients with ≥4 positive lymph nodes [123]). For both modalities, very high local control rates have been reported in non-randomised series [124–127] suggesting a clinical benefit of eliminating time for repopulation (‘temporal miss’) and reducing geographic miss. However, conclusions regarding potential superiority of an IORT boost over a conventional postoperative boost must await long-term follow-up of randomized trials [4].

The dose of RT that can be applied to inactivate residual tumour cells is limited by toxicity in the irradiated normal tissue. With Intrabeam, skin toxicity is avoided by keeping a distance >5 mm between the skin and the applicator surface [75]. Radiobiological modelling of late reaction suggested that pneumonitis is limited to distances ≤8–12 mm from the applicator surface so that the thickness of the thorax wall should be sufficient to shield the lung from the irradiation [70]. The risk of fibrosis in the subcutaneous tissue was estimated to be limited to 3–6 mm from the applicator surface [70, 128]. These estimates are likely to be further reduced by the volume effect of late-reacting normal tissues. Thus the tissue tolerance is increased when the volume exposed to critical doses is reduced [129, 130] although recent evidence suggests that the effect may be weak for breast fibrosis [131]. Pneumonitis has not been reported for the TARGIT trial but the ELIOT trial found less lung toxicity in the IOERT compared with the EBRT arm and similar rates of breast fibrosis in the two arms [123]. Overall, the rate of fibrosis after risk-adapted IORT with Intrabeam was similar to that after EBRT but subanalysis showed that it was associated with higher rates after IORT as a boost in combination with postoperative EBRT and lower rates after IORT alone [132]. Thus only 5.9% of patients treated with IORT alone developed clinical fibrosis of the breast at 36 months [132] consistent with the estimates from radiobiological modelling discussed above. The most frequent wound-related complications after IORT with Intrabeam are hematomas and seromas but the rate is not higher than after EBRT [78, 133].

For IORT given as a boost, previous work from our department showed that the risk of late reaction after EBRT is increased if the interval between surgery/IORT and EBRT is shorter than five weeks [75]. For IORT boost with LEX, moderate to severe fibrosis at 36 months follow-up was observed in 43 and 31% of the patients treated with an intervals shorter and longer than 5-week, respectively [132]. The latter value may be compared with rates of approximately 20% at 3 years, rising to 28.1% at 10 years and 30.4% at 20 years, in the EORTC boost trial [134–136] and with approximately 25% at 5 years for a boost in the 25 × 2 Gy control arm of the START pilot trial [131]. Although the fibrosis rates after an IORT boost appears somewhat increased relative to a standard fractionated EBRT boost of 16 Gy, it compares favourably with the higher rates of 40–55% observed after a boost dose of 26 Gy in the EORTC boost trial [136]. However, a direct comparison of an intraoperative versus conventional postoperative boost will only be possible after long-term follow-up of the ongoing randomised TARGIT B trial.

A potential concern for high single-dose irradiation is the risk of tissue necrosis and rib fracture. Fat necrosis after IORT for breast cancer is found by diagnostic imaging although it is usually non-symptomatic [137, 138]. Rib fracture has been described after IOERT but is avoided by introducing lead shielding of the ribs [139]. Brain necrosis can occur after high local doses, dependent on the irradiated volume [140, 141], and should be considered when applying IORT to the brain [142]. In studies with typical target volumes in stereotactic radiosurgery, the risk has been shown to be predicted by the volume receiving a dose ≥ 12 Gy (V_12Gy_) although, for larger target volumes, V_10Gy_ may be a better predictor [143–145]. Because of the strong increase in symptomatic radionecrosis with increasing volume, it was suggested that patients with V_10Gy_ > 10.5 cm^3^ or V_12Gy_ > 8 cm^3^ be considered for hypofractionated rather than single-dose treatment [143].

## Conclusions

**Fig. 4:**
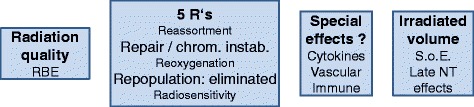
Schematic overview of biological effects contributing to the efficacy of IORT with high single doses. RBE: relative biologic effectiveness; S.o.E.: Sphere of Equivalence; NT: normal tissue

A schematic overview over the different biological aspects of IORT with high single doses is shown in Fig. 4. IORT is usually performed with different radiation qualities than high-energy X-rays used in conventional, fractionated RT. The increased RBE should be taken into account for LEX. Although the L-Q model predicts that RBE should decrease as the dose per fraction increases [20], evidence suggests that this may not be true for low-LET radiations. Conversely, this also implies that a slightly reduced RBE of high-energy electrons cannot be excluded even at high doses although this may be cell-type dependent. However, in clinical practice, RBE = 1 relative to high-energy X-rays is usually assumed for fractionated RT with electrons and more studies would be required to determine if a lower value should be applied for IOERT with single doses in the range of 10–20 Gy. A number of factors may contribute to making single-dose IORT biologically feasible in spite of being a departure from the established fractionated schemes based on the five R’s of radiotherapy. Published and presented evidence supports a hypothesis that saturation of the repair system leads to increasing genomic instability that may contribute to inactivate tumour cells as the dose per fraction is increased beyond the dose range normally studied in vitro. Furthermore, IORT performed during surgery eliminates repopulation of residual tumour cells in the tumour bed during the time for wound healing before starting and possibly during conventionally fractionated EBRT. Thus some patients are likely to have very few residual tumour cells which may be cured even by moderate doses to the tumour bed. In addition, the high dose close to the applicator surface of LEX is predicted to be more efficient than the uniform dose from an external beam, thus partly compensating the lower doses at larger depth in the tumour bed. Together with increased tolerance of the normal tissue to high local doses owing to the volume effect, the combination of these factors work in favour of making IORT more efficient than expected from clinical experience with EBRT. Whether special effects of high single doses also contribute to the efficacy will require further experimental and clinical studies.

## Additional files

Additional file 1: Supplementary Materials and Methods and Supplementary Results. (PDF 41 kb)

Additional file 2: Figures S1-S5. **Figure S1.** Survival curves for irradiation of cells (V79, MCF7, HUVEC) in vitro with 10 MeV electrons or 6 MV X-rays. **Figure S2.** A: Induction and decay of the mean number of γH2AX foci per cell (V79). **Figure S2.** B-E: Distributions of the number of foci in individual cells 30 min after irradiation. **Figure S3.** A, B: Sublinear dose response for the mean number of γH2AX foci per cell (MCF7) at maximum induction and after repair. **Figure S3.** C: The fraction of γH2AX foci remaining at 240 min (4h) in V79 as function of dose. **Figure S4.** Decay of γH2AX foci with increasing post-irradiation repair time. **Figure S5.** A, B: Split-dose recovery of cell survival of V79 and MCF7 cells. C, D: Surviving fractions as function of split-dose interval time. E, F: λ × t derived from Eq. (3) of the Supplementary Materials and Methods as function of split-dose interval. (PDF 229 kb)

Additional file 3: DSB and SLD repair data. (XLSX 26 kb)

Additional file 4: RBE and gH2AX data. (XLSX 56 kb)

## Abbreviations

alt-EJ: Alternative end joining
ASMase: Acid sphingomyelinase
CFA: Colony formation assay
CS: Ceramide synthase
DNA-PKcs: DNA protein kinase, catalytic subunit
DSB: Double-strand break
EBRT: External beam radiotherapy
ELIOT: Electron intra-operative radiotherapy trial
EQD2: Equivalent dose given in 2 Gy fractions
H2AX: Histone H2AX
HR: Homologous recombination
HUVEC: Human umbilical vein endothelial cells
IOERT: Intraoperative electron radiotherapy
IORT: Intraoperative radiotherapy
LET: Linear energy transfer
LEX: Low-energy X-rays
LIG1: Ligase 1
LIG3: Ligase 3
LIG4: Ligase 4
L-Q: Linear-quadratic
NF-κB: Nuclear factor kappa B
NHEJ: Non-homologous end joining
PARP1: Poly(ADP-ribose)-Polymerase 1
RBE: Relative biologic effectiveness
RT: Radiotherapy
SF: Surviving fraction
SLD: Sub-lethal damage
START: UK standardisation of radiotherapy trial
TARGIT: Targeted intraoperative radiotherapy trial
TEM: Transmission electron microscopy
WRN: Werner syndrome ATP-dependent helicase
XRCC1: X-ray repair cross complementing 1
XRCC4: X-ray repair cross complementing 4

## Footnotes

1 After acceptance of the present manuscript, an analysis of the effect of overall treatment time has been published by the START Trial Management Group [150]. Based on the combined cohorts of all three START trials (START pilot, START A, and START B), the effect of overall treatment time on loco-regional control was estimated at 0.60 Gy/day. Although independent validation of this observation is needed, the time effect is higher than the rough estimate made here suggesting that the potential benefit of IORT in eliminating the repopulation associated with fractionated WBRT might be even larger than 28 Gy.
