# Supplementary material for: Biology of high single doses of IORT: RBE, 5 R’s, and other biological aspects
Source: Radiat Oncol. 2017 Jan 19;12:24. doi: 10.1186/s13014-016-0750-3 (PMC5251326; doi:10.1186/s13014-016-0750-3)
Supplement: Supplementary file 1 — Supplementary Materials and Methods and Supplementary Results. (PDF 41 kb) [file 13014_2016_750_MOESM1_ESM.pdf]

Supplementary Materials and Methods

Cells lines and strains

The hamster lung fibroblast cell line, V79-4, was obtained from Prof. K.J. Weber, Laboratory of Molecular and Cellular Radiobiology, Department of Radiation Oncology and Radiotherapy, Radiological University Clinic, Heidelberg, Germany. These cells were cultivated in RPMI1640 medium supplemented with 10% fetal bovine serum (FBS; Biochrom AG, Berlin, Germany). The epithelial breast cancer cell line, MCF7, was a generous gift from Prof. H. Allgayer, Dept. of Experimental Surgery, Universitätsmedizin Mannheim, Germany, and was grown in Dulbecco's MEM supplemented with 10% FBS. Human umbilical vein endothelial cells (HUVEC) were isolated from umbilical cord obtained from the Maternity Department, Frauenklinik, Universitätsmedizin Mannheim, Germany. The cells were cultivated in Basal Endothelial medium (customer formulation; Promo-Cell, Heidelberg, Germany) with growth factors (supplement pack, Promo-Cell, Heidelberg, Germany) supplemented with 10% FBS. All cell types were grown as monolayer at 37°C in T25 flasks (Falcon, BD Biosciences, Heidelberg, Germany) under 5% CO_2 .They were subcultivated 2-3 times weekly by mild trypsination using a 1-2 fold concentrated Trypsin/EDTA solution (1-fold: 0.05% Trypsin (Biochrom AG, Berlin, Germany) and 0.02% EDTA in phosphate-buffered saline, PBS). Cultures of the V79 and MCF7 cell lines were used a maximum of 20 passages after which they were discarded and a new vial from the frozen stock of cells was thawed. HUVEC were used until passage 8.

Colony formation assay

Cellular radiosensitivity for inactivation of clonogenic proliferation was determined by the colony formation assay (CFA). Cells were seeded in triplicate in T25 flasks (Falcon, BD Bioscience) and incubated for 5 h to allow spreading before irradiation. Sham irradiated flasks were treated as the irradiated flasks. After irradiation, all flasks were incubated at 37°C with an atmosphere of 5% CO_2 for colony formation for 6 days (V79), 12 days (MCF7), or 10 days (HUVEC). After incubation, the colonies were fixed and stained as described (1). Clones containing at least 50 cells were scored as colonies, and the surviving fraction (SF) of colony-forming cells was plotted versus absorbed dose in a semilogarithmic diagram. A minimum of three independent repeat experiments were performed for each condition. The dose-response curves were fitted by the linear-quadratic model: $\ln[SF(D)] = -(\alpha D+\beta D^2)$. The RBE was calculated as the ratio of the dose of 6 MV X-rays (D_{ref}) and dose of 10 MeV electrons (D_{10MeV}) producing the same effect (SF): $RBE = D_{ref} / D_{10MeV}$.

Immunohistochemical detection of γH2AX foci

Exponentially growing cell cultures were seeded into 8-well chamber slides (Nunc Lab-Tek™, Thermo Fischer Scientific, Langenselbold, Germany). The cells were incubated for one day at 37°C under 5% CO_2 before irradiation. After irradiation, they were returned to the incubator for various post-irradiation times before fixation. The slides were washed once with PBS and cells fixed with 3.7% formaldehyde in PBS/T (0.2% Triton X-100 in PBS) for 10 min, and blocked with 1% BSA in PBS/T for 10 min at room temperature. The chamber slides were incubated with mouse monoclonal anti-γH2AX (anti-phospho-histone H2A.X (Ser139)) antibody (Millipore GmbH, Schwalbach/Ts, Germany) diluted with PBS/T (1:500) for 1 h at room temperature, then washed 10 × 2 min with PBS/T and subsequently incubated with secondary FITC-labeled anti-mouse-antibody (Chemicon International Deutschland, Hofheim, Germany) diluted with PBS/T (1:1000) for 1 h in the dark at room temperature. Cells were washed with PBS 10 times for 15 min, the plastic chambers were removed and a cover slip was applied using mounting medium consisting of 10% Mowiol 4-88 (Calbiochem, Merck KgaA, Darmstadt, Germany), 2% 1,4-Diacabicyclo-(2,2,2)-octan (Fluka, Sigma-Aldrich Chemie GmbH, Munich, Germany), 25% glycerol, and 0.1 M Tris-HCl, pH 8.5 (Sigma, Sigma-Aldrich Chemie GmbH, Munich, Germany). The cells were photographed under a fluorescence microscope (Leica TCS-SP2-XL, Leica Microsystems GmbH, Wetzlar, Germany) and images were acquired from randomly selected fields. Foci were scored in 50-120 cells and the mean number of foci per cell was calculated. Three independent experiments were performed.

Determination of halftimes for cell recovery (SLD repair)

In order to analyse recovery kinetics (SLD repair), the early time interval between two equal doses was varied from 5-40 min. The G-value for two acute doses, D_1 and D_2, separated by a time interval, t, can be calculated analytically. For total dose, $D=D_1+D_2$ and repair rate constant, λ , the following expression has been derived (2):

$$G = \frac{D_1^2 + D_2^2 + 2\, D_1\, D_2\, \exp(-\lambda t)}{D^2} \qquad \text{eq. (1)}$$

G is factored into the quadratic term of the L-Q model to account for SLD repair: $-\ln(SF) = \alpha D + G\beta D^2$. Thus by rearrangement, G can be expressed as

$$G = \frac{-\alpha D - \ln(SF)}{\beta D^2} \qquad \text{eq. (2)}$$

Eliminating G from these two equations allows calculation of λt. For two equal doses, $D_1 = D_2 = D/2$, the following expression for $\exp(-\lambda t)$ is derived

$$\exp(-\lambda t) = 2\frac{-\ln(SF)}{\beta D^2} - \frac{2(\alpha/\beta)}{D} - 1 \qquad \text{eq. (3)}$$

SF was plotted as function of the split-dose time interval in Figure 4.3.14a,b, and λt was calculated from eq. (3). The SLD repair rate constant, λ, may then be determined as the slope of a plot of λt as function of t.

Irradiation

10 MeV electrons from a linear accelerator were used to simulate IORT with electrons. Adherent cells were irradiated in T25 flasks at SSD=100 cm with 2 mm of medium. Additional buildup of 5 mm RW3 (Göttingen White Water; Physikalisch-Technische Werkstätten, PTW, Freiburg, Germany) and 10 mm PMMA (polymethylmethacrylate, 1.2 g/cm^3 density) was used to simulate 20 mm water-equivalent depth. The doses in the setting for cell irradiation were measured by GafChromic film dosimetry and yielded 95±3% of nominal dose at 20 mm equivalent depth (errors are estimated standard deviations (3). 6 MV X-rays were used as reference radiation with 15 mm water-equivalent material for maximum dose buildup (100%). Linear accelerators in the radiotherapy department were used (Siemens Mevatron in early experiments, Elekta Synergy in all subsequent experiments) at a dose rate of 4-6 Gy/min. Dosimetry was performed by the department medical physicists as part of the daily quality check.

Data analysis, statistics.

Linear-quadratic curve fitting of survival curves was performed by non-linear least-squares regression. For determination of RBE and the L-Q coefficients, α and β, data from individual experiments were fitted using the JMP statistical software package version 11 (SAS Institute Inc., Cary, NC). RBE values at different survival levels were determined in each experiment and statistical significance was tested using the paired t-test. The 1-tailed t-test was used because RBE<1 with increasing electron energy (mean energy of secondary electrons from 6 MV X-rays << 6 MeV), and the test was performed on logarithmic values of the doses (because $RBE=D_{ref}/D_{test}$, i.e. $\log(RBE)=\log(D_{ref})-\log(D_{test})$). For graphical presentation, a second-order polynomial was fitted to mean values of ln(SF) versus dose using the regression wizard tool of the SigmaPlot scientific graphics software Version 8.0 (SPSS Inc., Chicago, IL). Distributions of γH2AX foci numbers per cell were analysed by JMP. Bi-exponential and

hyperbolic fitting of γH2AX foci decay was performed by SigmaPlot version 12.5 (Systat Software Inc., Erkrath, Germany). P-values <0.05 were considered significant.

Supplementary data

Irradiation of V79 cells with 0-11.4 Gy of 10 MeV electrons or 0-12 Gy of 6MV X-rays suggested an RBE value slightly less than unity at the highest dose (Supplementary Figure S1A. In order to validate this and to test if the RBE might decrease further at higher doses, further experiments were performed at 15-18 Gy X-rays (14.3-17.1 Gy electrons). This confirmed the trend yielding RBE=0.94±0.2 (P=0.04) at SF=0.0003. MFC7 cells showed no significant difference in the initial series for doses up to 8.6 Gy (Supplementary Figure S1B) which was confirmed when the dose range was extended to 11.4 Gy (electrons) (shown in Figure 1A). A slightly reduced RBE was suggested for HUVEC in the dose range 5-6 Gy (Supplementary Figure S1C) which became significant when the dose range was extended to 8.6 Gy (shown in Figure 1B).

Irradiation with low-to-moderate doses induces rapid formation of γH2AX foci with a maximum at approximately 30 min followed by decay at later times (Supplementary Figure S2A). A sub-linear increase in the number of γH2AX foci at doses higher than 2-3 Gy (see main section) might conceivably result from overlapping foci imposing an upper limit for detection of individual foci due overlap of foci at high density. However, the distribution of the number of foci per cell would be expected to be skewed towards high numbers as the number of foci approached the maximum number which can be resolved which clearly was not the case for V79 and MCF7 cells (Supplementary Figure S2B-E). Furthermore, the change in slope would be predicted to be observed at a certain number of foci per cells. Thus after repair, this number should be the same and the dose at which it is reached should be higher than at maximum induction (30 min). In fact, deviation from linearity of γH2AX foci versus dose occurred at a much lower mean number of foci but at approximately the same dose in V79 (shown in Figure 2A), and in MCF7 and HUVECs (Supplementary Figure S3A,B).

In V79 cells, the fraction of radiation-induced γH2AX foci remaining at 240 min after irradiation increased significantly in the dose range 0.95-5.7 Gy (Supplementary Figure S3C). A further change in the high-dose region (5.7-17.1 Gy) was not significant, which was also confirmed in MCF7 and HUVECs at 360 and 480 min respectively. In the high-dose region, the decay kinetics could be fit by a biexponential function (Supplementary Figure S4A-F)

consistent with the fast and slow components of DSB rejoining detected by physical methods after high doses (4, 5). However, because this model has four free parameters the uncertainty on the estimates was large. A hyperbolic fit representing reciprocal kinetics as proposed by Fowler (6) has only two parameters and gives more robust fit parameters at the expense of a slightly poorer fit. The fraction of induced foci remaining was used as a surrogate for the composite decay up to 6-8h and showed no significant difference in the rate of repair between the two doses although a constant decay in terms of numbers of foci independent of dose could not be excluded.

Split-dose recovery was demonstrated for V79 and MCF7 cells (Supplementary Figure S5A,B). SLD repair kinetics was determined for irradiation of V79 with 6Gy+6Gy and MCF7 cells with 4Gy +4Gy with split-dose intervals up to 40 min. Assuming that only the fast component was important, early halftimes were determined (see Supplementary materials and methods and Supplementary Figure S5C-F).

Supplementary References

1. Liu Q, Schneider F, Ma L, Wenz F, Herskind C. Relative Biologic Effectiveness (RBE) of 50 kV X-rays Measured in a Phantom for Intraoperative Tumor-Bed Irradiation. Int J Radiat Oncol Biol Phys. 2013;85(4):1127-33.
2. Sachs RK, Hahnfeld P, Brenner DJ. The link between low-LET dose-response relations and the underlying kinetics of damage production/repair/misrepair. Int J Radiat Biol. 1997;72(4):351-74.
3. Schneider F, Polednik M, Wolff D, Steil V, Delana A, Wenz F, et al. Optimization of the gafchromic EBT protocol for IMRT QA. Z Med Phys. 2009;19(1):29-37.
4. Dikomey E, Franzke J. Effect of heat on induction and repair of DNA strand breaks in X-irradiated CHO cells. Int J Radiat Biol. 1992;61(2):221-33.
5. Iliakis G, Wang H, Perrault AR, Boecker W, Rosidi B, Windhofer F, et al. Mechanisms of DNA double strand break repair and chromosome aberration formation. Cytogenetic and genome research. 2004;104(1-4):14-20.
6. Fowler JF. Is repair of DNA strand break damage from ionizing radiation second-order rather than first-order? A simpler explanation of apparently multiexponential repair. Radiat Res. 1999;152(2):124-36.
