# Supplementary material for: Biology of high single doses of IORT: RBE, 5 R’s, and other biological aspects
Source: Radiat Oncol. 2017 Jan 19;12:24. doi: 10.1186/s13014-016-0750-3 (PMC5251326; doi:10.1186/s13014-016-0750-3)
Supplement: Supplementary file 2 — Figures S1-S5. Figure S1. Survival curves for irradiation of cells (V79, MCF7, HUVEC) in vitro with 10 MeV electrons or 6 MV X-rays. Figure S2. A: Induction and decay of the mean number of γH2AX foci per cell (V79). Figure S2. B-E: Distributions of the number of foci in individual cells 30 min after irradiation. Figure S3. A, B: Sublinear dose response for the mean number of γH2AX foci per cell (MCF7) at maximum induction and after repair. Figure S3. C: The fraction of γH2AX foci remaining at 240 min (4h) in V79 as function of dose. Figure S4. Decay of γH2AX foci with increasing post-irradiation repair time. Figure S5. A, B: Split-dose recovery of cell survival of V79 and MCF7 cells. C, D: Surviving fractions as function of split-dose interval time. E, F: λ × t derived from Eq. (3) of the Supplementary Materials and Methods as function of split-dose interval. (PDF 229 kb) [file 13014_2016_750_MOESM2_ESM.pdf]

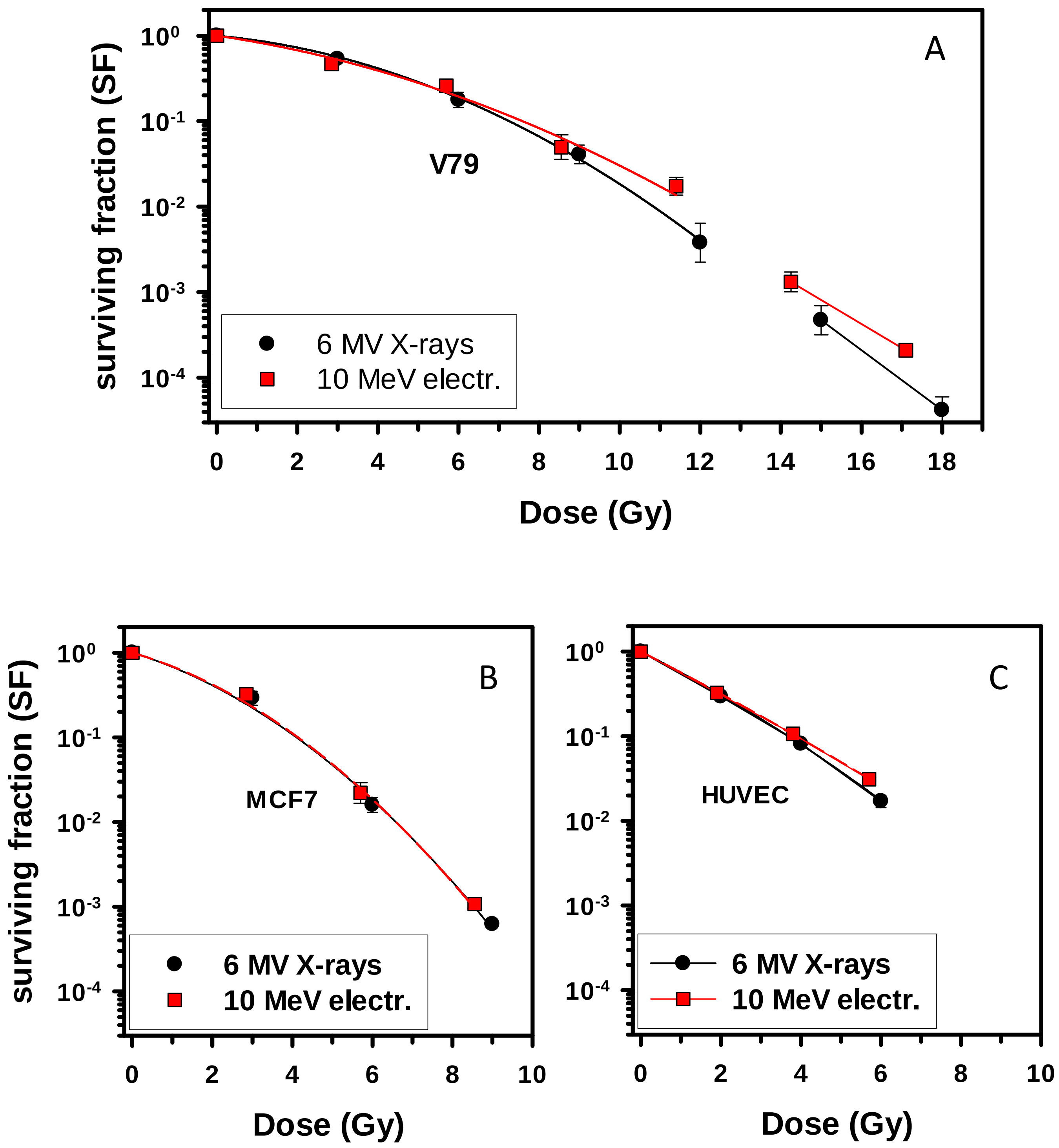

Supplementary **Figure S1**. Survival curves for irradiation of cells *in vitro* with 10 MeV electrons (20 mm water-equivalent depth) or 6 MV X-rays. For V79 cells, in total n=7 independent experiments were performed (n=4 for 3-12 Gy and and n=3 for 14-18 Gy) (A). Trends for reduced RBE of electrons were observed for V79 cells (RBE=0.89±0.04, P=0.04, n=4 at SF=0.02, RBE=0.94±0.02, P=0.04, n=3 at SF=0.0003) (A) and HUVEC (RBE= 0.92±0.03; P=0.07, n=3 at) (C). No significant difference was observed for MCF7 cells (RBE=1.00±0.01, P=0.41, n=2-3) (B). Mean values and std.err. are shown.

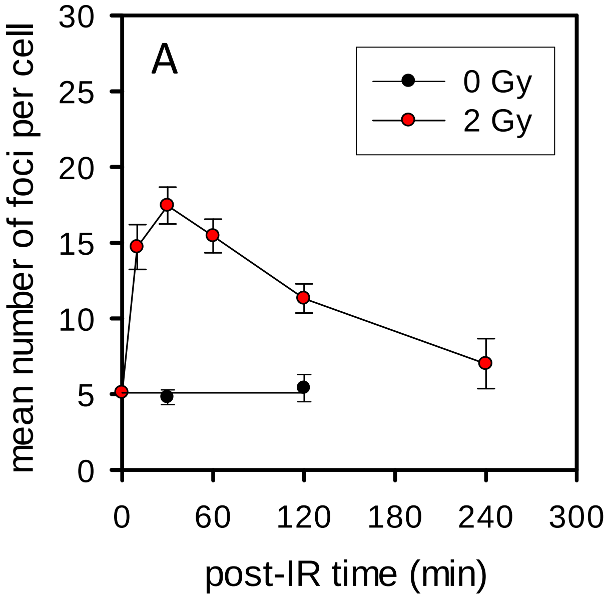

Supplementary **Figure S2A**. Induction and decay of the mean number of γH2AX foci per cell (V79) after a dose of 2 Gy of 6 MV X-rays (corresponding to a single fraction of conventional external beam radiotherapy). Mean values and std.err. are shown

Supplementary **Figure S2B-E**. Distributions of the number of foci in individual cells 30 min after irradiation. No indication was observed that the upper detection limit for detection of the number of γH2AX foci was reached after moderate to high doses. Thus the distributions of the number of foci in individual cells were not skewed towards higher numbers in V79 cells after 17.1 Gy (C) versus 5.7 Gy of 10 MeV electrons (B), or in MCF7 cells after 8 Gy (E) vs 4 Gy (D) of 6 MV X-rays.

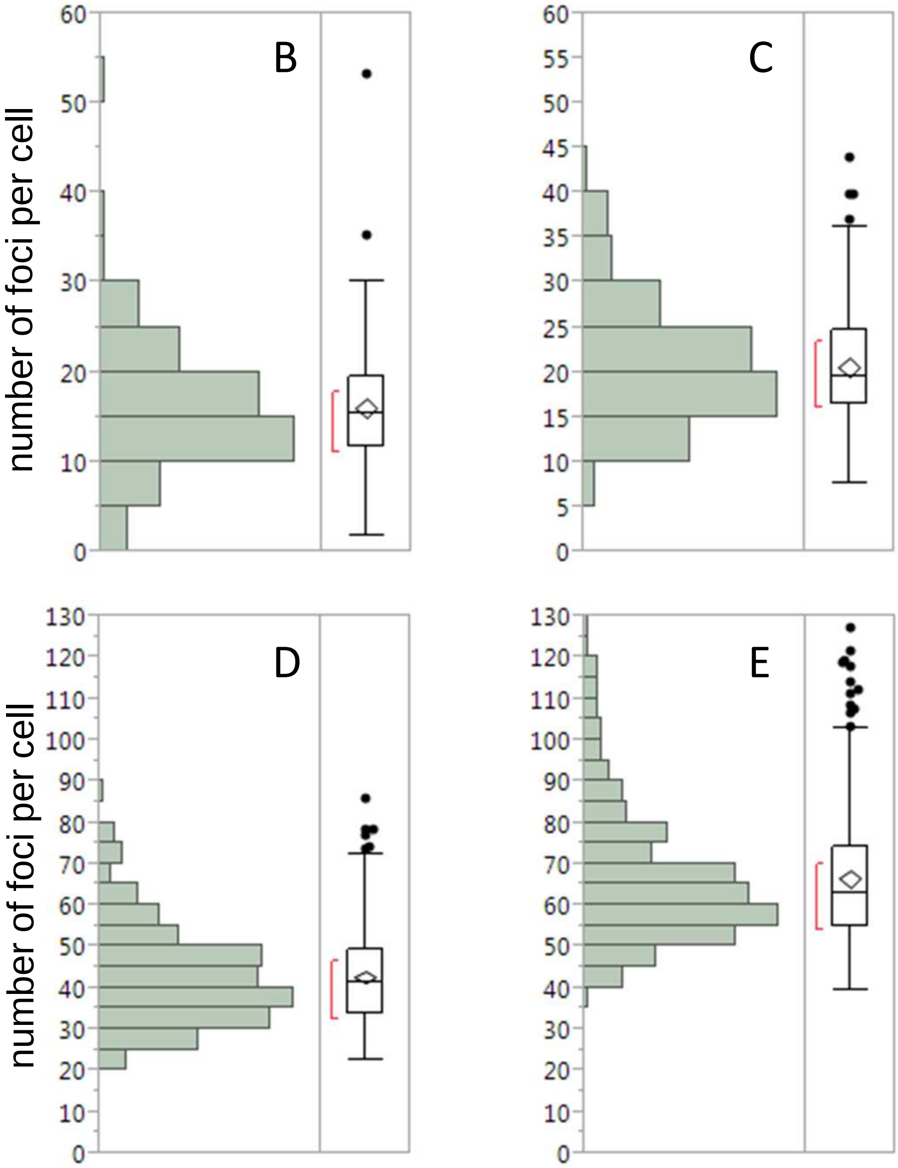

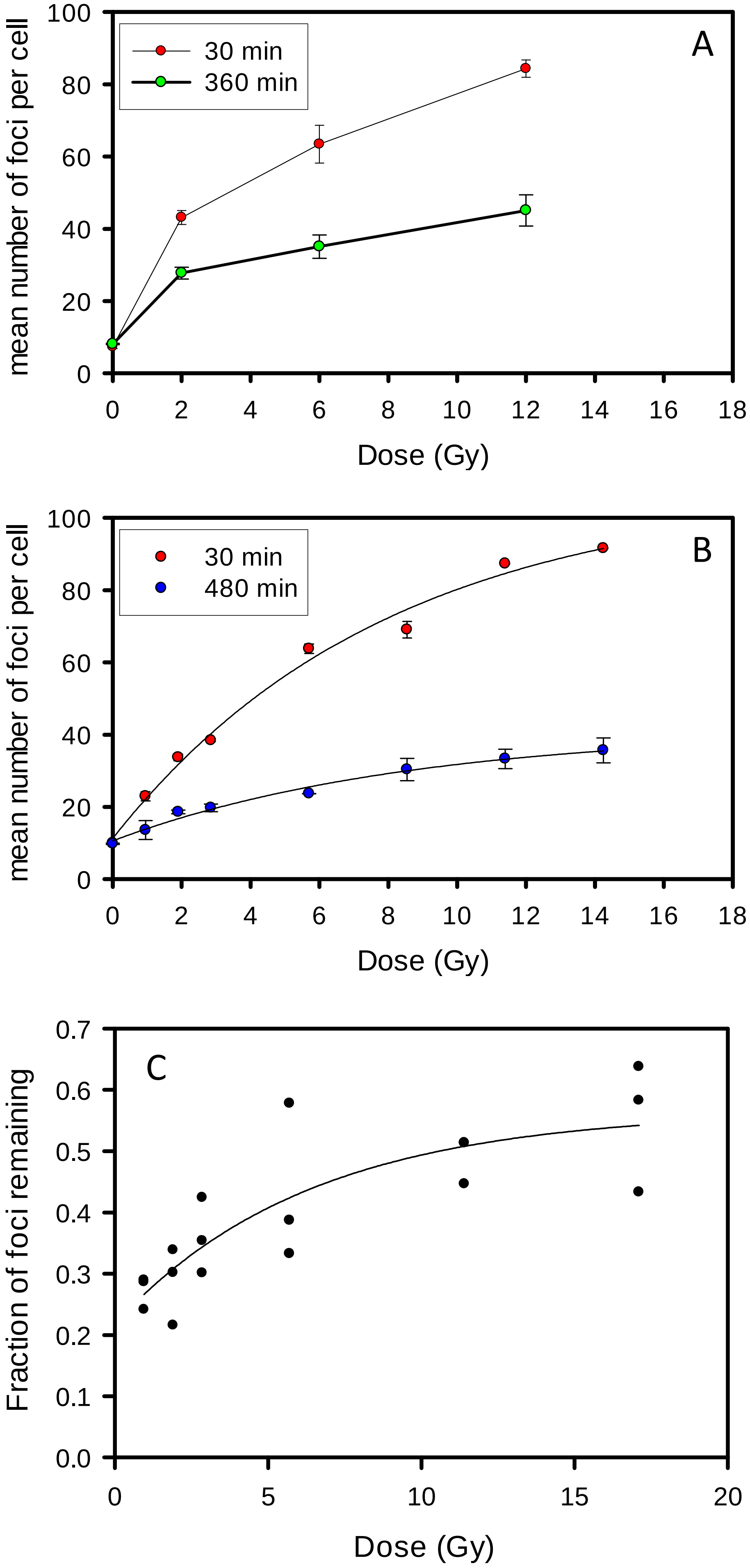

Supplementary **Figure S3**.

A: Sublinear dose response for the mean number of γH2AX foci per cell (MCF7) at maximum induction and after repair (30 min and 360 min (6h) post-irradiation, respectively). 6 MV X-rays. Mean values and std. err. are shown (n=3).

B: Sublinear dose-response the mean number of γH2AX foci per cell (HUVEC) at maximum induction and after repair (30 min and 480 min (8h) post-irradiation, respectively). 10 MeV electrons. Mean values and std. err. are shown (n=3).

C: The fraction of γH2AX foci remaining at 240 min (4h) in V79 cells increased significantly as function of dose in the range 0.95-5.7 Gy (10 MeV electrons). Data points from three independent experiments are shown.

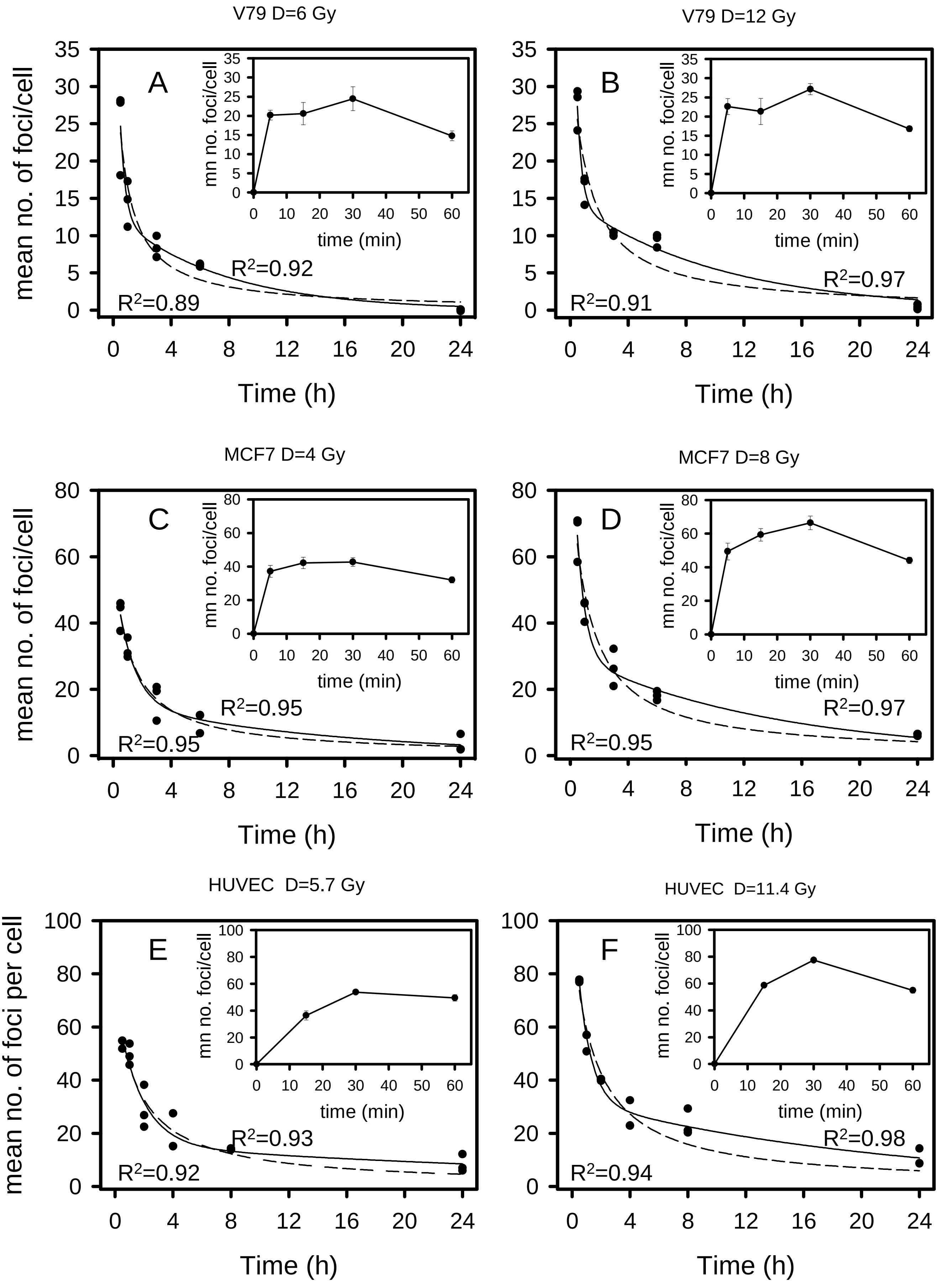

Supplementary **Figure S4**. Decay of γH2AX foci with increasing post-irradiation repair time. Solid curves represent least-squares fits of bi-exponential decay, broken curves represent hyperbolic least-squares fits. A-D: V79 and MCF7 cells irradiated with 6 MV X-rays; E, F: HUVECs irradiated with 10 MeV electrons (20 mm water-equivalent depth). Induction at early time points is shown as inserts. Mean values and std.err. are shown (n=3).

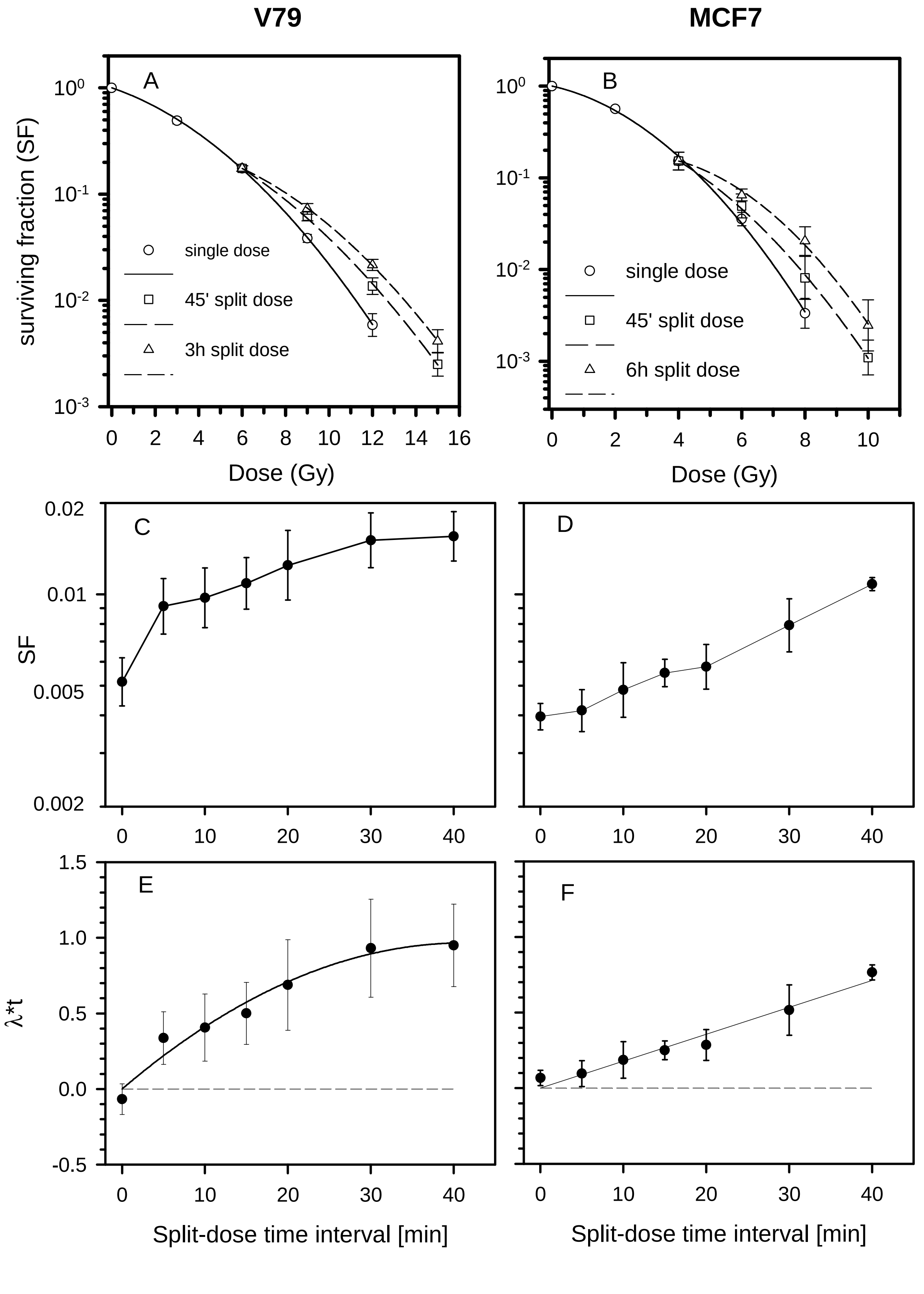

Supplementary Figure S5. A, B: Split-dose recovery of cell survival for irradiation of V79 cells with 6 Gy, and MCF7 cells with 4 Gy of 6MV X-rays, followed by graded doses after different time intervals. C, D: Surviving fractions as function of split-dose interval time (5-40 min) for irradiation of V79 cells with 6 Gy + 6 Gy and MCF7 cells with 4 Gy + 4 Gy. E, F: $\lambda \times t$ derived from Eq. (3) of the Supplementary data as function of split-dose interval. The rate constant, λ,for SLD repair is equal to the slope. Mean values and std. err. are shown (n=3).
